# Supplementary material for: Uniaxial and Coaxial Electrospinning for Tailoring Jussara Pulp Nanofibers
Source: Molecules. 2021 Feb 24;26(5):1206. doi: 10.3390/molecules26051206 (PMC7956372; doi:10.3390/molecules26051206)
Supplement: Supplementary file 1 [file molecules-26-01206-s001.pdf]

**Supplementary Materials:**

**Table S1.** Effect of the estimates for the diameter of the fibers (nm) containing anthocyanins from jussara pulp using the CCDR.

| Variable | Coefficient | p-value |
|---|---|---|
| Mean | 1489.33 | 0.0004 |
| $X_1$ (PEO) | 178.09 | 0.1680 |
| $X_2$ (NaCl) | 69.27 | 0.6210 |
| $X_1$ (PEO) | 627.02 | 0.0024 |
| $X_2$ (NaCl) | 627.02 | 0.0037 |
| $X_1$ (PEO) x $X_2$ (NaCl) | 1364.00 | 0.0003 |

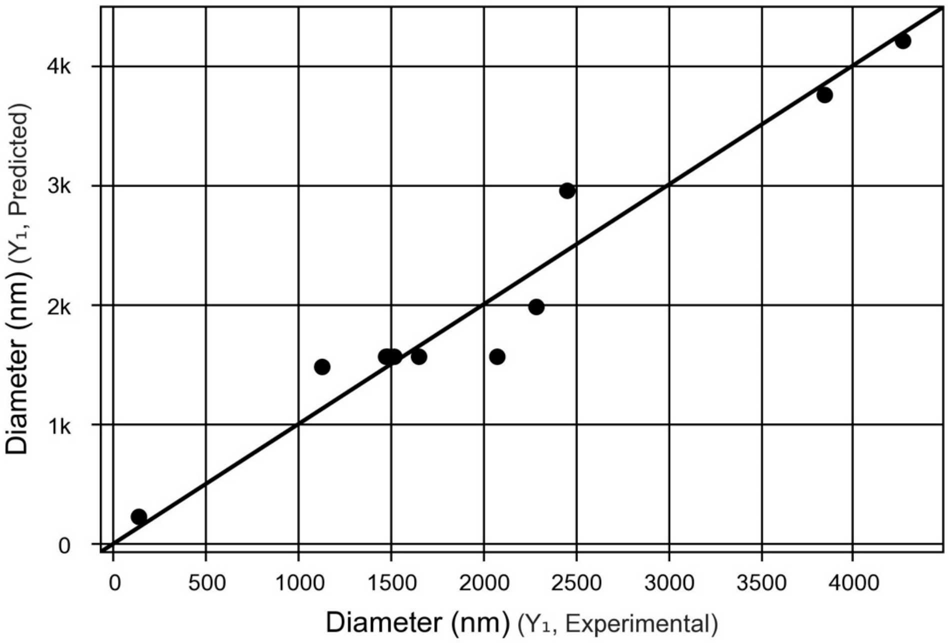


**Figure S1**. Correlation between the experimental and predicted values of the diameter fiber from the model equation of the CDDR.
